# Characterization and Childhood Tumor Risk Assessment of Genetic and Epigenetic Syndromes Associated With Lateralized Overgrowth

**DOI:** 10.3389/fped.2020.613260

**Published:** 2020-12-17

**Authors:** Jessica R. Griff, Kelly A. Duffy, Jennifer M. Kalish

**Affiliations:** ^1^Division of Human Genetics, Children's Hospital of Philadelphia, Philadelphia, PA, United States; ^2^Departments of Genetics and Pediatrics, Perelman School of Medicine, University of Pennsylvania, Philadelphia, PA, United States

**Keywords:** lateralized overgrowth, hemihypertrophy, hemihyperplasia, Beckwith-Wiedemann spectrum, Beckwith-Wiedemann syndrome (BWS), PIK3CA-related overgrowth spectrum (PROS), Proteus syndrome (PS), PTEN hamartoma tumor syndrome

## Abstract

Lateralized overgrowth (LO), or segmental overgrowth, is defined as an increase in growth of tissue (bone, muscle, connective tissue, vasculature, etc.) in any region of the body. Some overgrowth syndromes, characterized by both generalized and lateralized overgrowth, have been associated with an increased risk of tumor development. This may be due to the underlying genetic and epigenetic defects that lead to disrupted cell growth and proliferation pathways resulting in the overgrowth and tumor phenotypes. This chapter focuses on the four most common syndromes characterized by LO: Beckwith-Wiedemann spectrum (BWSp), *PIK3CA*-related overgrowth spectrum (PROS), Proteus syndrome (PS), and *PTEN* hamartoma tumor syndrome (PHTS). These syndromes demonstrate variable risks for tumor development in patients affected by LO, and we provide a comprehensive literature review of all common tumors reported in patients diagnosed with an LO-related disorder. This review summarizes the current data on tumor risk among these disorders and their associated tumor screening guidelines. Furthermore, this chapter highlights the importance of an accurate diagnosis when a patient presents with LO as similar phenotypes are associated with different tumor risks, thereby altering preventative screening protocols.

## Introduction

**Figure 1:**
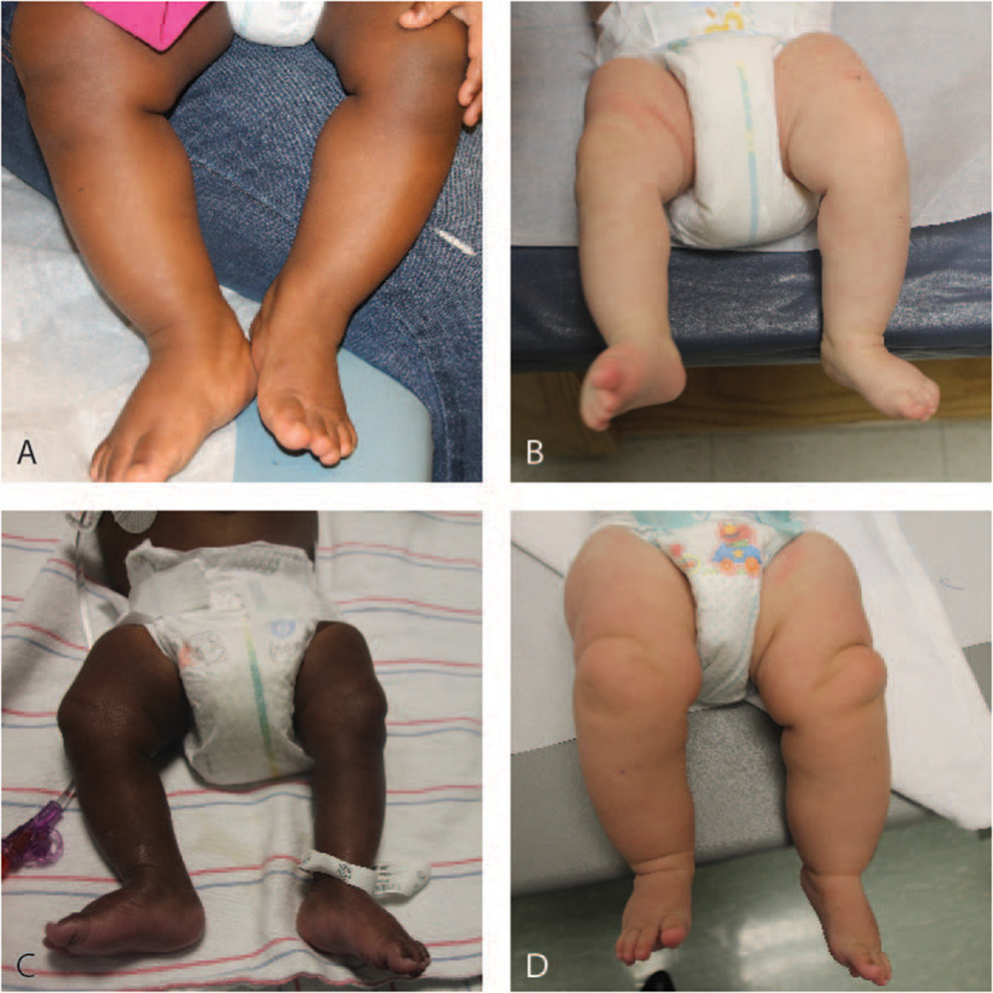
Legs of patients with lateralized overgrowth. **(A)** 12-month old patient with Beckwith-Wiedemann syndrome. **(B)** 3-month old patient with Beckwith-Wiedemann syndrome. **(C)** 9-month old patient with Beckwith-Wiedemann syndrome. **(D)** 6-month old patient with PIK3CA-related overgrowth spectrum.

Lateralized overgrowth (LO) is defined as any type of segmental overgrowth (1) (Figure 1). The nomenclature was developed to classify patients who were previously described with overgrowth due to both hyperplasia (OMIM 235000), a proliferation of cells, and hypertrophy (OMIM 235000), an increase in cell size. The overgrowth defined by LO is not specific to the type of tissue affected and can include skeletal, muscular, adipose, and/or vascular tissues. Some patients present with isolated LO, in which patients are primarily affected by LO. Overgrowth of organs is not required for the designation of LO, but it can be present and typically occurs in patients with overgrowth syndromes associated with LO.

Patients with isolated lateralized overgrowth (ILO), those affected by LO but lacking other features and patterns of malformations, dysplasia, and morphologic variants, have been reported to have an increased development of tumors, primarily the embryonal tumors Wilms tumor (WT) and hepatoblastoma (HB) (2, 3), similar to the most common tumor types observed among patients affected by LO and overgrowth disorders (4). A prior study of patients with isolated hemihypertrophy, now referred to as ILO, reported 9 out of 168 developing a tumor (5) and two cases of HB in patients with isolated hemihyperplasia, now also termed ILO (6). Retrospectively, it is likely that many of these patients could be classified with an overgrowth or cancer predisposition syndrome.

There are several genetic and epigenetic syndromes associated with LO and ILO. These molecular changes may influence the tissue type, location of the observed overgrowth, and associated tumor risk in patients. In this chapter, we review the clinical characteristics of the most common genetic and epigenetic syndromes associated with LO. We focus on tumor development and risks associated within each syndrome and summarize current screening recommendations.

Common considerations for all suspected LO-related overgrowth disorder include the underlying molecular cause and appropriateness for tumor surveillance.

### Molecular Considerations

The underlying mechanisms for the disorders described are complex and beyond the focus of this review. A brief description of the currently understood mechanisms for each disorder is summarized and includes both genetic and epigenetic mechanisms. One consideration for molecular investigation for these disorders is that some defects can present as mosaic, in which the proportion of normal cells to cells with the molecular change varies in any given tissue, leading to patients with somatic molecular defects. This means that positive molecular detection may only be found in affected tissue(s), whereas blood sample analyses may yield negative results. Other patients affected by LO and overgrowth have the molecular defect change(s) detectable in blood samples (constitutional defects), with some patients affected by changes that are inheritable or considered germline defects.

### Tumor Risk and Screening

Specific recommendations and implementation of tumor surveillance protocols are determined by the risk of tumor development in a particular syndrome, the uniformity of the tumors that develop (i.e., can they be screened for in a non-invasive manner), and the health care environment in which the screening is occurring (i.e., the threshold of acceptable risk) (4). In some syndromes with an established tumor risk, tumor screening has been demonstrated to detect tumors at an early age. For example, in Beckwith-Wiedemann spectrum (BWSp), patients who underwent ultrasonographic screening had on average earlier tumor stages at diagnosis than those who did not undergo screening (7). Diagnosing tumors in their earlier stages may allow for less invasive treatment and the prevention of possible metastasis.

Here, we review the most common syndromes characterized by LO: BWSp (OMIM 130650), *PIK3CA*-related overgrowth spectrum (PROS), Proteus syndrome (PS) (OMIM 176920), and *PTEN* hamartoma tumor syndrome (PHTS). Tumor development in these four syndromes is variable and discussed below.

## Beckwith-Wiedemann Spectrum (OMIM 130650)

### Overview

BWSp is the most common and well-characterized overgrowth and cancer predisposition disorder and is caused by a variety of molecular defects in the chromosome 11p15 region. The disorder is estimated to affect 1 in 10,340 live births and disproportionately affects patients conceived by assisted reproduction techniques, estimated to affect 1 in 1,100 live births (8, 9). The clinical manifestations and subsequent phenotype of patients with BWSp can be highly variable, leading to the reclassification of the disorder from a syndrome [Beckwith-Wiedemann syndrome (BWS)] to a spectrum [BW spectrum (BWSp)] by an international consensus group (10). The consensus group created a clinical scoring system to guide molecular and clinical diagnosis. They classified features as those classically associated with the disorder (cardinal features) and features associated with the disorder but that can also occur in the general population (suggestive features). This scoring system was implemented to determine if genetic testing is necessary (10). Cardinal features include macroglossia, omphalocele, muscular LO, bilateral WT, hyperinsulinism, adrenal cytomegaly, pancreatic adenomatosis, and placental mesenchymal dysplasia, and suggestive features include macrosomia, facial nevus simplex, polyhydramnios or placentomegaly, ear creases or pits, transient hypoglycemia, embryonal tumors, nephromegaly and/or hepatomegaly, and umbilical hernia or diastasis recti. Each cardinal feature receives two points, and each suggestive feature receives one point. A total clinical score greater or equal to 2 indicates the need for genetic testing for BWSp. A clinical score greater or equal to 4 (typically including at least one cardinal feature) is sufficient for a clinical diagnosis of BWSp even if no molecular defect on chromosome 11p15 is identified. Genetic testing is also recommended for patients with a family history of BWSp caused by a heritable alteration.

### Molecular Considerations

BWSp is caused by a variety of genetic and epigenetic alterations in the BWS critical region on chromosome 11p15.5 (10). The BWS critical region contains two imprinted regions, which control the normal regulation of fetal and postnatal growth genes through a process called methylation. The majority of patients are affected by abnormal methylation in the imprinting control 1 (IC1) and/or imprinting control 2 (IC2) regions, with the most common cause being loss of methylation at KCNQ1OT1:TSS DMR (IC2 LOM) (~50% of patients) (10). Other causes of BWSp include paternal uniparental isodisomy of chromosome 11p15 (pUPD11), gain of methylation at H19/IGF2:IG DMR (IC1 GOM), mutations of *CDKN1C*, and other genetic aberrations including deletions, duplications, and translocations that affect chromosome 11p15 (10).

### Tumor Risk in BWSp

The risk for WT, HB, and neuroblastoma in BWSp is well documented (11–18). A patient's tumor risk varies based on the molecular etiology of BWSp. According to the recent international consensus for BWSp, for patients with IC1 GOM, the overall risk of tumor development is 28%, and the risk for WT is 24%. For patients with IC2 LOM, the overall tumor risk is 2.5%. For patients with pUPD11, the overall tumor risk is 16%. The risk for developing a WT is 8%, and the risk for developing a HB is 3.5%. Screening guidelines are constantly evolving based on ongoing research on this topic and are dependent on geographical location and cultural context of clinical practice. The European guidelines include abdominal ultrasounds every 3 months until the age of 7 years for patients with BWSp due to IC1 GOM, pUPD11, *CDKN1C* mutations, and other chromosome aberrations of the BWS region (10). The United States guidelines developed by the American Association for Cancer Research (AACR) Childhood Predisposition Workshop include abdominal ultrasounds and alpha-fetoprotein (AFP) screening every 3 months until the 4th birthday and renal ultrasounds every 3 months from the 4th to the 7th birthday for all patients with BWSp (4). In addition, patients with *CDKN1C* mutations, those at the highest risk for developing a neuroblastoma among patients with BWSp, should receive urine vanillylmandelic acid (VMA), homovanillic acid (HVA), and chest X-rays screening every 3 months until the 6th birthday and every 6 months from the 6th to the 10th birthday (10). Patients with BWSp caused by genome-wide paternal isodisomy (GWpUPD) have been reported to have additional tumors and beyond these screening windows. Patients with this molecular subtype should be monitored closely (10, 19).

## *PIK3CA*-Related Overgrowth Spectrum

### Overview

The phenotypic variety and overlap of individual syndromes caused by *PIK3CA* mutations prompted the establishment of the term PROS (20). The specific overgrown tissue observed in patients with PROS is typically adipose or vascular; however, muscular and skeletal overgrowth has also been observed (20). Other common clinical characteristics include epidermal nevus, macrodactyly, hemimegalencephaly (HMEG), seborrheic keratoses, and benign lichenoid keratoses (20). To determine the eligibility for genetic testing, clinical characteristics are divided into two categories: category A, which includes a spectrum of overgrowth, vascular malformations, and epidermal nevus phenotypes, and category B, which includes isolated features, such as lymphatic malformations or macrodactyly. Genetic testing is warranted if a patient presents with two or more features from category A, or one feature from category B, that was/were congenital or developed during early childhood.

A diagnosis of PROS is confirmed with a pathogenic variant found in the *PIK3CA* gene; however, if a mutation is not detected, the patient retains a clinical diagnosis of PROS if the clinical criteria are met (20, 21). In patients affected by clinical diagnoses of PROS, it is likely that the negative genetic result(s) observed are due to the somatic and therefore mosaic nature of the *PIK3CA* mutation leading to the phenotype, which may be difficult to detect from a single sample (such as blood).

### Molecular Considerations

*PIK3CA* is a protein coding gene for p110α that is the α subunit of a collection of catalytic subunits for phosphatidylinositol 3-kinase (PI3K) (22). This protein is important for regulating signals for cell proliferation and survival. Mutations in *PIK3CA* have been identified as the driver for many cancers in asymptomatic patients (those without phenotypes related to *PIK3CA* abnormalities), with common cancer types including breast (>30%), endometrial (>30%), bladder (>20%), colorectal carcinoma (>17%), and head and neck squamous cell carcinoma (>15%) (23).

*PIK3CA* mutations have also been identified in patients with the following syndromes: fibroadipose overgrowth (FAO) (24), congenital lipomatous overgrowth, vascular malformations, epidermal nevi, scoliosis/skeletal and spinal (CLOVES) syndrome (25, 26), megalencephaly-capillary malformation (MCAP) syndrome (27), Klippel-Trenaunay syndrome (KTS) (26, 28), and HMEG (29). Typically, *PIK3CA* mutations occur post-fertilization (somatic mutations), but there have been germline *PIK3CA* mutations reported (30, 31). Allelic heterogeneity in PROS (and other overgrowth disorders) and the overlap of common variants in the genes responsible may influence cancer predisposition, but further study is required.

### Tumor Risk in PROS

Tumor risk and surveillance for patients with PROS is currently debated. Gripp et al. suggested similar screening guidelines for patients affected by PROS to the guidelines for patients affected by ILO or BWS, which includes abdominal ultrasounds until the 7th birthday (32). Peterman et al. suggest sonographic screening for patients with CLOVES, MCAP, and diffuse capillary malformations only if LO is present (33, 34). To our knowledge, there have been 12 patients with PROS reported with malignant or potentially malignant renal findings [including WT, nephrogenic rests (NR), and indeterminate WT/NR findings]. NR and nephroblastomatosis (NBL) are capable of transforming into WT, but they are not tumors themselves (35, 36). Among the PROS patients with renal findings, eight patients with findings reported had a molecularly confirmed PROS diagnosis: four reported with WT development, two with reported indeterminate WT/NR findings, and two with NR (26, 28, 32, 37–40). Four additional patients with renal findings and without molecular PROS confirmation have also been reported (41–44). Postema et al. estimated the tumor risk between 1 and 2%, suggesting that under European standards, screening is not warranted (39); however, at that risk level by US guidelines, screening would be warranted (4).

As the focus of this review is to discuss common syndromes associated with LO and tumor screening guidelines, determining the true WT risk in PROS is beyond the scope of this chapter. A meta-analysis of PROS patients and WT development is currently being performed and will be reported separately in the literature once completed. Based on the current literature, the risk depends on how the reported cases are classified (for example, true WT vs. those with indeterminant malignant potential, such as NBL and NR). The tumor risk in the PROS population appears to be slightly less than what Postema et al. reported (~1–2%), and therefore it is unclear whether screening is warranted. The AACR tumor screening guidelines suggest screening when the risk of developing cancer is 1% or greater (4). It is suspected that the total patients with *PIK3CA* mutations currently classified may be higher than reports suggest (due to difficult detection of low levels of mosaicism). If this is true, the number of patients affected by PROS with tumors and the associated tumor risk for this disorder are likely well below the 1% threshold to warrant screening. Additionally, through our experience and discussions with colleagues, we are aware of many unreported patients with molecularly confirmed PROS who have not developed a WT or NR. We suspect that it is likely that the overall risk falls below 1%, indicating that screening is not warranted. It is also possible there are more patients with PROS and NR that have not been reported, as the NR did not progress to NBL or WT requiring treatment. There is a clear need for further publication of known cases and collaboration among institutions, so the denominator of patients with PROS can be further adjusted to understand true WT risk in this population. In terms of current recommendations, tumor screening should be performed at the discretion of the provider based on the genetic change and clinical features of the PROS presentation, as well as the family perspective.

In addition to WT and NR, there are four case reports of patients with PROS who developed other cancers including leukemia, vestibular schwannoma, retinoblastoma, and a meningioma (45–47); however, these do not suggest a specific predisposition or warrant surveillance.

### Additional Considerations

Studies on cell-free DNA of urine of patients with PROS found *PIK3CA* mutations in urine samples of patients who developed renal abnormalities, but not in patients with PROS who did not have a history of kidney irregularities (48, 49). As a result, it has been suggested that urine may be useful in detecting *PIK3CA* mutations, and those patients with positive results in urine may represent an increased risk for WT development (48). There may be other specific circumstances that could increase tumor risk, such as known *PIK3CA*-related changes in proximity to the kidneys or patients affected by specific germline or somatic mutations, but further study using larger cohorts is needed to better understand mechanisms and individual risk.

## Proteus Syndrome (OMIM 176920)

### Overview

PS is caused by postzygotic *de novo* activating mutations in *AKT1* (50). Clinical features of the syndrome include asymmetric skeletal growth, connective tissue nevi, epidermal nevi, vascular malformations, and dysregulated adipose tissue (lipomas, lipohyperplasia, fatty overgrowth, and partial lipohyperplasia) (51). Overlapping disorders, such as CLOVES, under the umbrella of PROS prompted the creation of a new diagnostic scoring system for PS (52). Five points are attributed for cerebriform connective tissue nevus, disproportionate overgrowth, and organ/visceral overgrowth. Two points are attributed for bullae or cysts of the lungs, dysregulated adipose tissue, linear verrucous epidermal nevus, vascular malformations, deep vein thrombosis/pulmonary embolism, and certain facial features, such as dolichocephaly and a low nasal bridge. Single points are attributed for specific tumors including genital cystadenomas, parotid monomorphic adenoma, and meningiomas (52). Points are subtracted for features, such as substantial prenatal extracranial overgrowth and ballooning overgrowth (52).

A diagnosis is confirmed if a patient has a score of 15 or more regardless of the presence of an *AKT1* variant. A patient with 10 or more points with an identified mosaic *AKT1* variant is considered to have PS. Those with scores between 2 and 9 points with an AKT1 variant are considered to have *AKT1*-related overgrowth spectrum (AROS) (52).

### Molecular Considerations

The *AKT1* gene located on chromosome 14q.32.33 is involved in the mTOR pathway that is responsible for regulating cell proliferation and survival (50). Patients with PS have a somatic, activating mutation in this gene that causes the observed abnormal growth. This mutation is not found in blood cells, and therefore a biopsy of the affected skin or tissue is required for a molecular diagnosis.

### Tumor Risk in PS

There are currently no tumor screening guidelines for patients with PS. However, a variety of benign and malignant tumors have been reported. Common neoplasms in patients with PS include lipomas, hamartomas, and vascular malformations (53). There have been multiple reports of patients with PS who developed genital cysts as well as meningiomas (52–55). Other case reports of benign tumors include an optic nerve tumor, pinealoma, monomorphic parotid adenoma, intraductal papilloma, goiter, leiomyomas, papillary adenoma of appendix testis, papillary adenoma of kidney, and epibulbar tumor (53, 54, 56–58). Malignant tumors in patients with PS have also been reported. They include papillary thyroid carcinoma, mesothelioma of tunica vaginalis and peritoneal surface, intraductal carcinoma of the breast, endometrial cancer, ovarian carcinoma, and paratesticular ovarian-type papillary serous carcinoma (53, 54, 59–64).

Early mortality in patients with PS is high yet does not appear to be related to the development of cancer (65), as pulmonary embolisms, postoperative embolisms, and pneumonia are responsible for mortality in 20% of patients with PS (51). It is possible that tumor risk is higher in this population, especially benign tumors, but due to the high mortality, an increased tumor risk is not observed.

## *PTEN* Hamartoma Tumor Syndrome

### Overview

PHTS is the umbrella term for genetic syndromes caused by germline *PTEN* mutations. Common clinical features of pediatric patients with PHTS include macrocephaly, hamartomas, lipomas, cardiac defects, and autism (66). LO is due to adipose and vascular anomalies. Major and minor criteria were implemented to aid in diagnosis. Major criteria include the presence of macrocephaly, macular pigmentation of the glans penis, and multiple mucocutaneous lesions, and minor criteria include autism, lipomas, and vascular malformations (66, 67).

### Molecular Considerations

*PTEN* is a tumor suppressor gene on chromosome 10q23 and is also involved in the mTOR signaling pathway (68). Germline mutations of *PTEN* cause PHTS and have been identified in patients with Cowden syndrome and Bannayan–Riley–Ruvalcaba syndrome (69). There have also been case reports of patients with an initial clinical diagnosis of PS, but a *PTEN* mutation was identified, leading to the term Proteus-like syndrome (70–72).

### Tumor Risk in PHTS

The tumor risk in patients with PHTS is well-documented although the syndrome is not typically associated with early childhood cancer risks. Tumors tend to develop in females more than males. The cumulative cancer risk by age 50 for females is 81% and for males is 48% (73).

Malignant tumors commonly observed in patients with PHTS include breast (25–50%), thyroid (3–17%), endometrium (9–27%), melanoma (1–6%), renal (4–16%), and colorectal cancers (3–13%) (73–82). Lhermitte-Duclos disease (LDD) also known as gangliocytoma of the cerebellum is common to develop late in life in patients with germline *PTEN* mutations (83, 84). Common benign tumors including hamartomas and lipomas can develop in patients at any age and require attention (evaluation and work-up) because of secondary complications that can arise.

There is no international consensus for tumor screening protocols in PHTS. In pediatric patients with PHTS, annual thyroid ultrasounds for thyroid cancer surveillance are recommended although the age to initiate surveillance is debated. The National Comprehensive Cancer Network (NCCN) guidelines for pediatric patients with PHTS include annual thyroid ultrasounds at the time of diagnosis, but Schultz et al. suggest starting ultrasounds at age 7 since the youngest reported case of thyroid cancer in a patient with PHTS was 7 years old (85, 86). In adult patients, colorectal screening beginning at age 40 is recommended (87), and the NCCN guidelines outline additional cancer surveillance recommendations in adults with PHTS.

## Discussion

**Figure 2:**
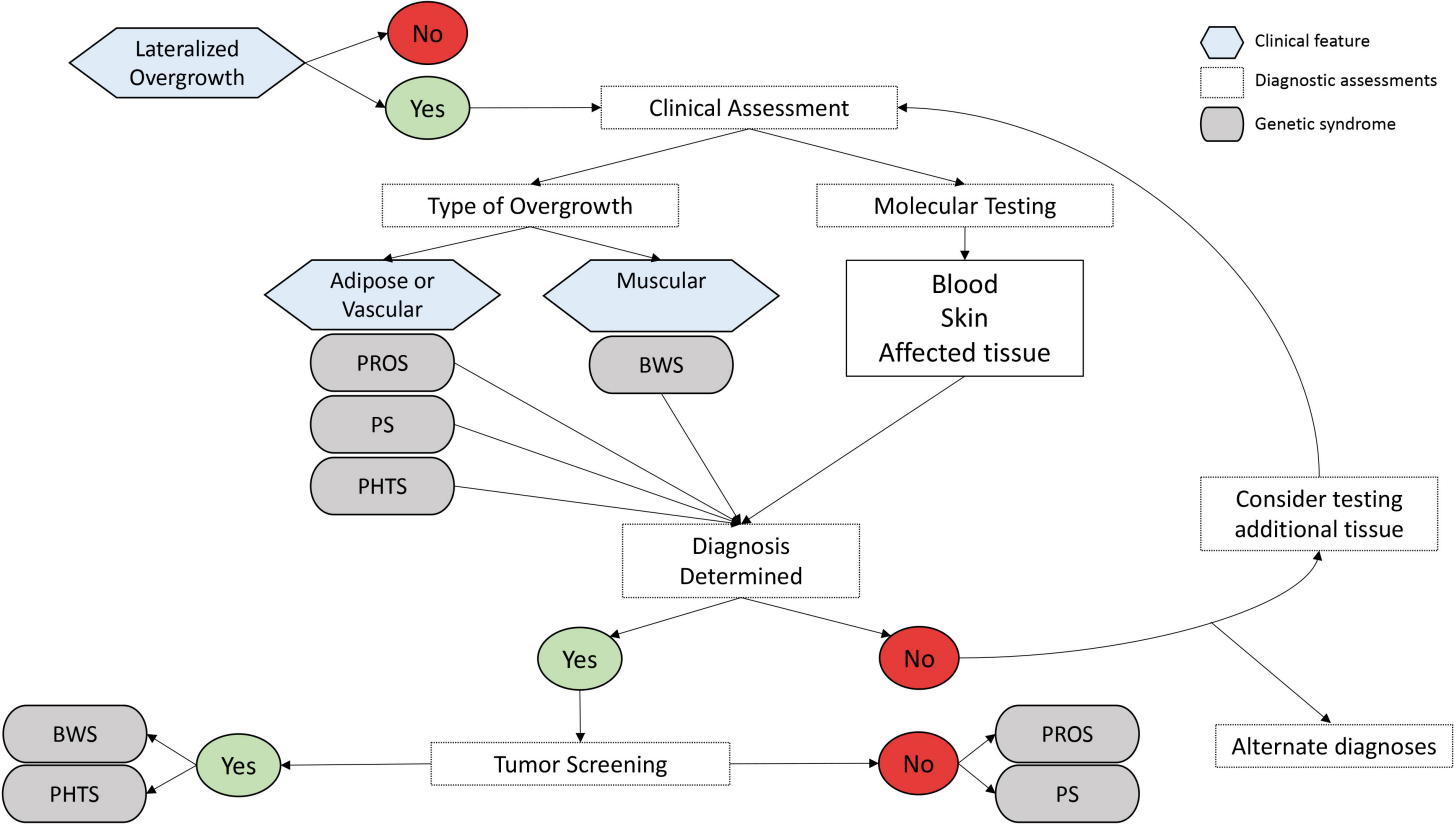
Differential diagnosis flowchart of lateralized overgrowth.

Narrowing the differential diagnosis and attaining confirmatory molecular testing results are critical for patient care management related to LO (Figure 2). The most common disorders and syndromes leading to LO have many overlapping clinical characteristics, making genetic testing useful for determining the underlying mechanism for the observed phenotype. For instance, PHTS is caused by a germline mutation (i.e., the genetic defect is present in every cell of the body), whereas PROS is mostly due to somatic alterations of the *PIK3CA* gene, leading to a mosaic distribution of the genetic defect throughout the body (i.e., some positive and negative cells). It is suspected that certain regions of the body are more likely to develop tumors if that region contains the genetic defect. If the genetic defect is widespread as it is in germline mutations and constitutional defects, it is logical that the tumor risks may be higher; however, further research is needed to explore this hypothesis.

From this review, it is evident that there are drastic differences in tumor risks for patients with syndromic LO, some of which warrant childhood tumor surveillance programs and others that do not seem to contribute an increased tumor risk as part of the phenotype (Table 1). It is therefore of utmost importance to correctly diagnose these patients, so they can receive proper screenings and care. Patients with ILO due to increased muscle bulk but without an identifiable genetic cause are now included under the BWSp umbrella and should undergo routine screening like other patients with BWS (17). Given that the guidelines are still being developed for PROS, a discussion with the family about the risk is recommended. In LO disorders with increased tumor risks, the effectiveness of tumor screening goes beyond diagnosing tumors at earlier stages. One study found that parents of patients with elevated tumor risks prefer screening because when educated about their child's risk, it reduced their worry and psychological stress (88).

**Table 1:** Summary of tumor risks in genetic and epigenetic syndromes with lateralized overgrowth.

	**Genetic cause**	**Type of overgrowth**	**Malignant tumors**	**Tumor risk**	**Childhood surveillance recommendation(s)**
PROS	*PIK3CA* mutations^*^	Adipose, vascular	Wilms tumor	~1%	None (to be determined)
BWSp	Genetic and epigenetic alterations on chromosome 11p15.5	Muscular	Wilms tumor Hepatoblastoma (Neuroblastoma)	0.2–24% 0–3.5% 0.5–4.2%	Abdominal ultrasound and AFP screening every 3 months until the 4th birthday and renal ultrasounds from the 4th until the 7th birthday
PS	*AKT1* mutations	Skeletal, adipose, vascular	None	Unknown	None
PHTS	*PTEN* mutations	Adipose, vascular	Breast Thyroid Endometrium Melanoma Kidney Colorectal	25–50% 3–17% 9–27% 1–6% 4–16% 3–13%	Annual thyroid ultrasounds beginning at the time of diagnosis

* *Majority are somatic mutations, but there have been case reports of patients with germline PIK3CA mutations*.

Overall, syndromes involving LO are heterogenous both within a given syndrome and between syndromes. As a result, tumor risk across the spectrum of LO disorders varies greatly due to the underlying cause of the syndrome, as well as personal tumor risk due to specific abnormalities present. Therefore, following diagnostic criteria to diagnosis, each patient will aid in assessing his/her individualized tumor risk and screening program.

## Author Contributions

JG performed the literature search and drafted the manuscript and figures. KD helped conceptualize the project, assisted with literature search, and edited the manuscript. JK conceptualized, organized, and edited the manuscript. All authors contributed to the article and approved the submitted version.

## Conflict of Interest

The authors declare that the research was conducted in the absence of any commercial or financial relationships that could be construed as a potential conflict of interest.
